# Drug-Induced Acute Eosinophilic Pneumonia With Pneumomediastinum: An Unusual Presentation

**DOI:** 10.7759/cureus.64708

**Published:** 2024-07-17

**Authors:** Vasu Agarwal, Sidhaant Nangia, Shaily Prasenan, Siri Vineeth A Ganta

**Affiliations:** 1 Respiratory Medicine, Dr. D.Y. Patil Medical College, Hospital and Research Center, Pune, IND; 2 Pulmonology and Critical Care, Dr. D.Y. Patil Medical College, Hospital and Research Center, Pune, IND

**Keywords:** pneumomediastinum, hypersensitive reaction, bronchial asthma exacerbation, complementary alternative medicine, drug induced eosinophilic pneumonia

## Abstract

A 27-year-old female, with no significant past medical history, presented to the casualty department with a two-week history of progressive dyspnea, cough, and fever. She reported that she had recently started taking a non-conventional alternative medication for her irregular menstrual cycles. Chest radiography demonstrated bilateral alveolar opacities, and computed tomography (CT) of the chest revealed bilateral ground-glass opacities and pneumomediastinum. Laboratory testing showed peripheral blood eosinophilia, and bronchoscopy with bronchoalveolar lavage confirmed an elevated eosinophil count. Based on the clinical presentation, radiographic and laboratory findings, and exclusion of other etiologies, a diagnosis of drug-induced eosinophilic lung disease with pneumomediastinum was made. The alternative non-conventional drug was immediately discontinued and the patient was treated with systemic corticosteroids, leading to a rapid improvement in her symptoms and radiographic abnormalities. A repeat CT of the chest after 15 days revealed significant resolution of the ground-glass opacities and complete resolution of pneumomediastinum. This case highlights the importance of thorough medication history and vigilance for potential adverse effects of non-conventional treatments.

## Introduction

Eosinophilic pneumonia is a rare disease caused by smoking [1], hypersensitivity reaction to drugs like antibiotics and non-steroidal anti-inflammatory drugs (NSAIDs) [2], parasitic infestations [3], and viral illnesses [4]. Drug-induced eosinophilic pneumonia manifests as acute or chronic pneumonia, accompanied by a history of drug intake and clinical improvement upon drug cessation. This is believed to be caused by a hypersensitivity reaction to the drug, leading to an eosinophilic lung infiltration with alveolar lung infection [1]. In our case, the patient had consumed non-conventional alternative medicine a few days before her presentation to the hospital with both acute eosinophilic pneumonia (AEP) and pneumomediastinum. There is insufficient literature to show a causal link between AEP and pneumomediastinum. The uniqueness of the case lies in the fact that soon after the drug consumption, the patient presented with both clinical disorders simultaneously. The drug-induced hypersensitivity reaction may have led to bronchial constriction and alveolar damage, causing an air leak from the alveoli into the mediastinum. Since the symptoms are vague, AEP diagnosis necessitates peripheral blood eosinophilia, bronchoalveolar lavage (BAL) eosinophilia, or a lung biopsy showing eosinophilic infiltration in the lung [5-8]. Pneumomediastinum, on the other hand, is mostly diagnosed radiologically [9]. The treatment revolves around corticosteroids for AEP [7] and symptomatic management for pneumomediastinum [10].

## Case presentation

**Figure 1:**
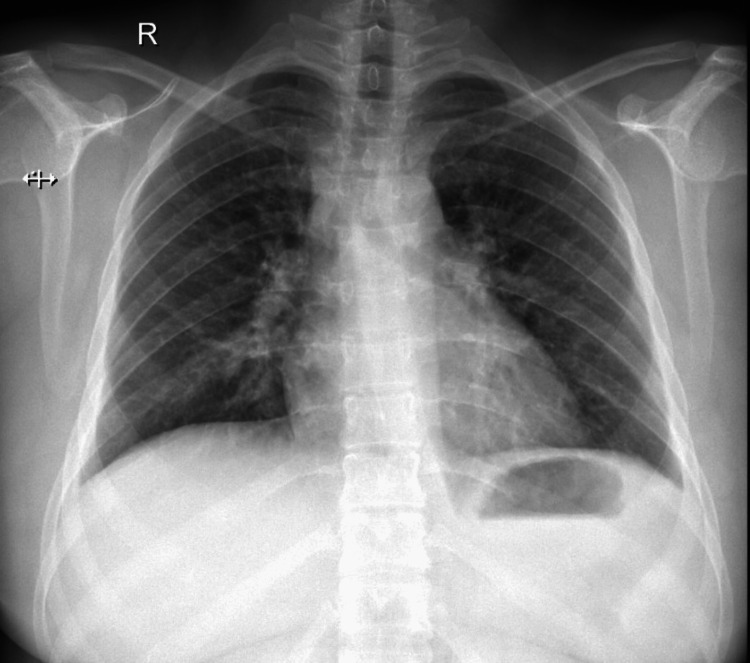
Plain chest radiograph with no apparent abnormalities

A 27-year-old female with no significant past medical history presented to our casualty with complaints of progressive breathlessness, dry cough, and fever for the last two weeks. The patient was tachycardic and tachypnoeic and had bilateral polyphonic rhonchi on lung auscultation. A subcutaneous emphysema in the neck was also detected, which was a surprising presentation because she had no history of any trauma, surgical procedures, mechanical ventilation, tracheostomy, or central venous catheter insertion, which might have caused the subcutaneous emphysema. The initial working diagnosis was of an acute infective exacerbation of bronchial asthma along with pneumomediastinum. She was subsequently admitted to the general ward and initiated on intravenous broad-spectrum antibiotics, nebulization with bronchodilators for the bronchoconstriction, inhaled corticosteroids along with intravenous systemic corticosteroids, and highly concentrated oxygen for the subcutaneous emphysema. The chest radiograph had no apparent lung parenchymal involvement (Figure 1).

**Figure 2:**
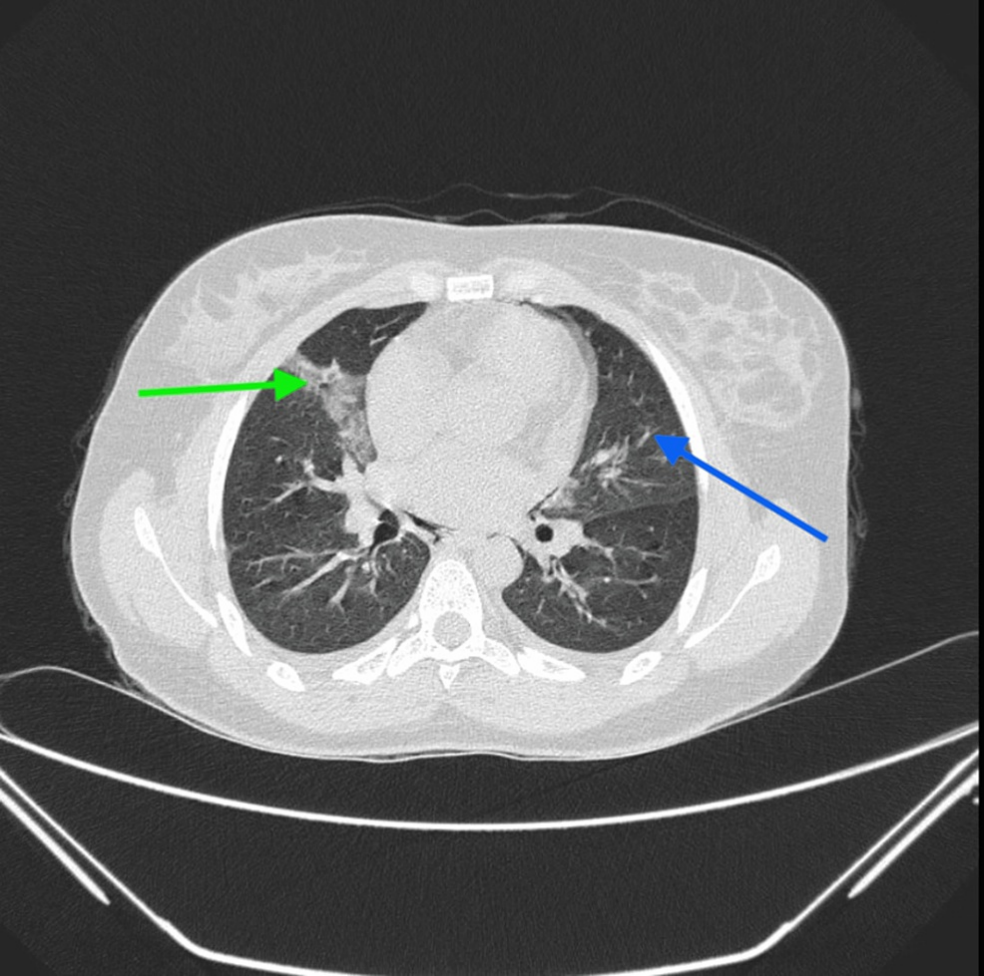
High-resolution computed tomography (HRCT) thorax with ground-glass opacities in the anterior and medial basal segments of the right lower lobe (green arrow) and the anteromedial segment of the left lower lobe (blue arrow)

**Figure 3:**
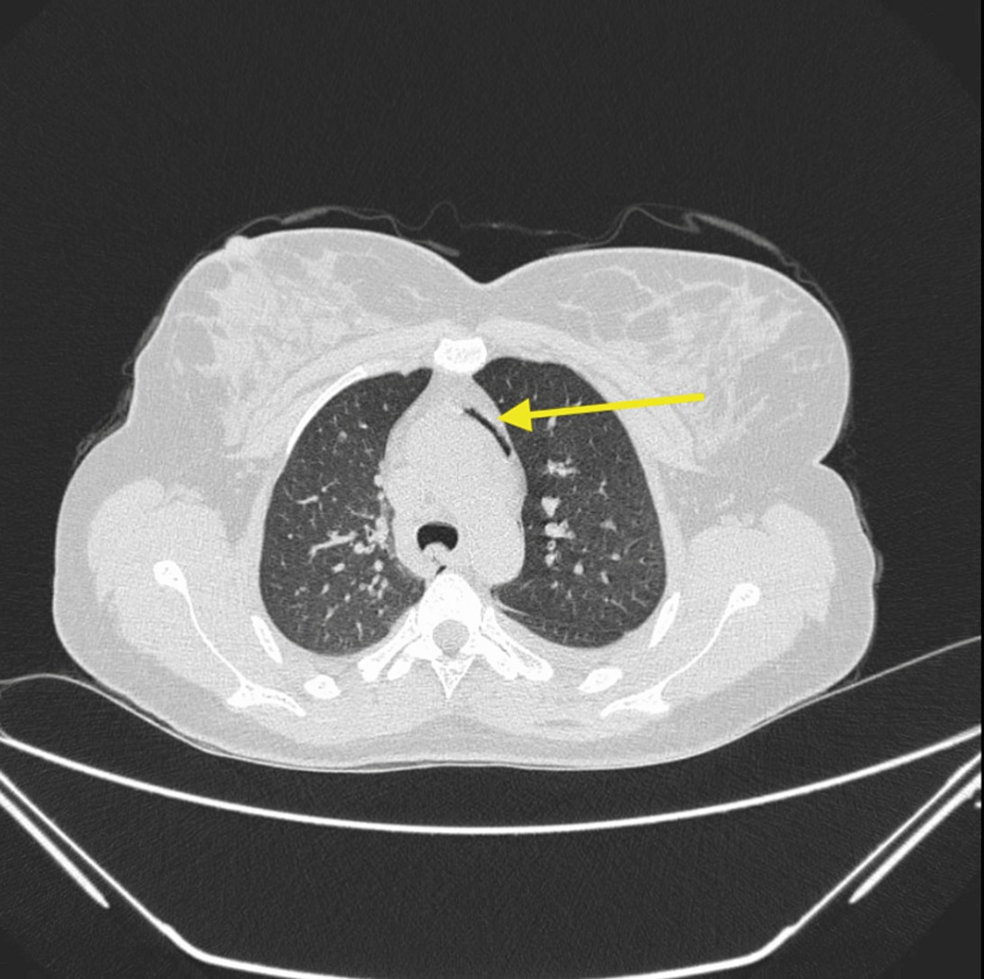
High-resolution computed tomography (HRCT) thorax with pneumomediastinum in the neck (yellow arrow)

A high-resolution computed tomography (HRCT) scan of the thorax showed mild bilateral pleural effusion with bilateral ground-glass opacities and a pneumomediastinum (Figures 2, 3).

A routine blood test showed a raised total leukocyte count suggestive of infection and peripheral blood eosinophilia, around 11%, with an absolute eosinophilic count of 1551 cells/microliter above the normal limits (Table 1).

**Table 1:** Complete blood count showing eosinophilia and raised absolute eosinophil count in peripheral blood (in bold) EDTA: ethylenediaminetetraacetic acid

Complete Blood Count (EDTA Whole Blood)	Result	Biological Reference Interval
Hemoglobin (Hb)	11.90	11.6 - 15.0 g/dL
Total leucocyte (WBC) count	14,100	4000-10000/µL
Platelet count	171,000	150000 - 410000 /µL
Red blood cell (RBC) count	4.55	3.92 - 5.13 x 10^6 /µL
Neutrophils	51	40 - 80 %
Absolute neutrophils	7,191	2000 - 7000 /µL
Eosinophils	11	1 – 6 %
Absolute eosinophils	1,551	20 - 500 /µL
Basophils	0	0 – 2 %
Absolute basophils	0	0 - 100 /µL
Lymphocytes	30	20 – 40 %
Absolute lymphocytes	4,230	1000 - 3000 /µL
Monocytes	8	2 - 10 %
Absolute monocytes	1,128	200 - 1000 /µL

**Figure 4:**
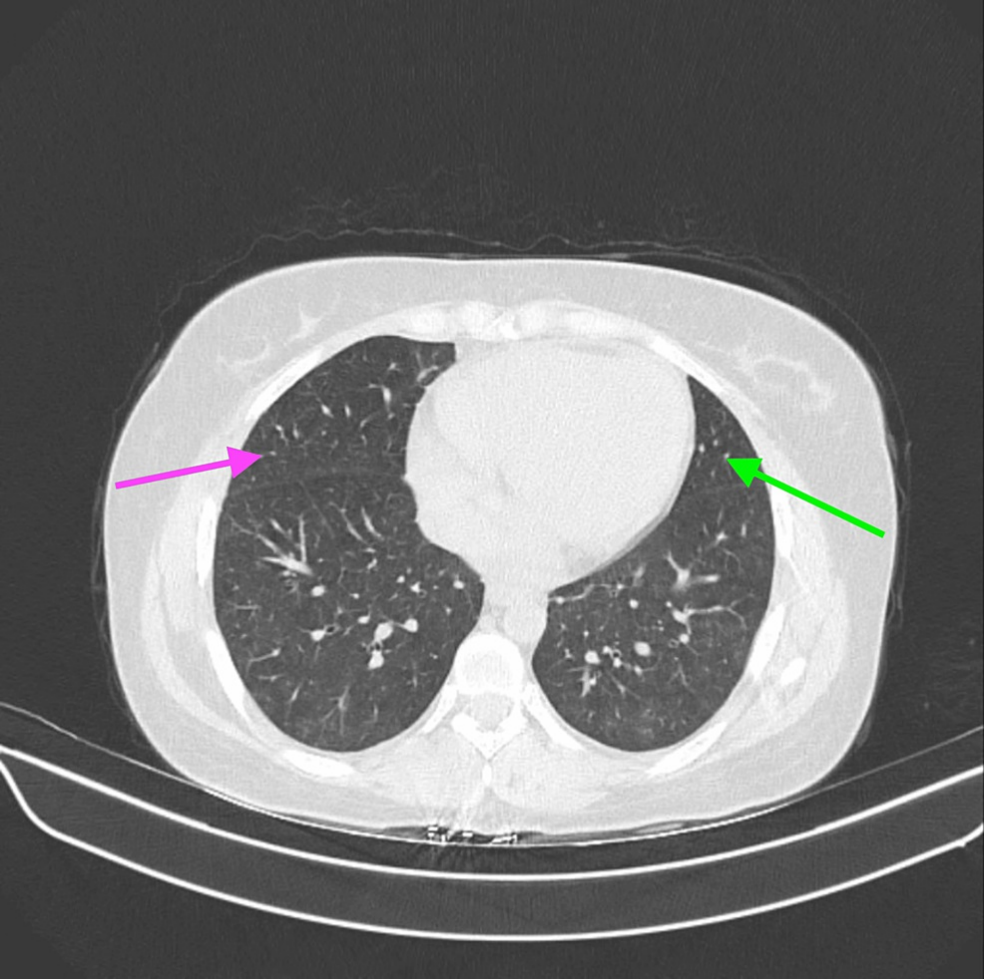
High-resolution computed tomography (HRCT) thorax showing a resolution of ground-glass opacities in the right lung parenchyma (pink arrow) and left lung parenchyma (green arrow)

Eosinophilia was unexplained, as the patient had no history of atopy, had not eaten any food from outside the home, had not traveled recently, and had no exposure that might have caused a parasitic infestation leading to eosinophilia. On thorough history-taking, the patient reported recent consumption of a non-conventional alternative medicine for her irregular menstrual cycles. Because the medication was a non-conventional drug therapy, it was impossible to determine its contents. We sent a serum immunoglobulin E assay, which came back significantly elevated above the normal limits. Suspecting a diagnosis of acute eosinophilic pneumonia, possibly due to drug consumption, we performed a bronchoscopy, and significant eosinophilia (more than 5%) was detected in the bronchoalveolar lavage (BAL). BAL cultures, as well as blood and urine cultures, were negative for microbial growth; ruling out an alternative explanation to her underlying pneumonia. We continued the patient on broad-spectrum intravenous antibiotics as prophylaxis, nebulized bronchodilators for her bronchoconstriction, and intravenous corticosteroids for AEP. We stopped the non-conventional alternative medication immediately and after a few days, the patient showed clinical improvement in symptoms and resolution of subcutaneous emphysema. The patient was successfully discharged and later on in the follow-up, the repeat HRCT thorax showed significant resolution of ground-glass opacities and a complete resolution of pneumomediastinum (Figure 4).

## Discussion

Acute eosinophilic pneumonia (AEP) is a rare disease, most commonly affecting younger men with a history of smoking [1]. Other causative factors include drug-induced eosinophilia [2], recreational use of inhalation drugs like cocaine [11] and marijuana [12], parasitic [3], fungal [13], and viral [4] infections. The development of eosinophilic lung disease in this patient was likely a consequence of the alternative non-conventional medicine she had recently started taking. Eosinophilic lung disease has been reported as an adverse effect of various conventional medications, including antibiotics, antidepressants, and nonsteroidal anti-inflammatory drugs [2]. However, it is an uncommon complication of complementary and alternative medicines. Although the pathogenesis remains incompletely understood, it could potentially involve a hypersensitive immune response by alveolar macrophages to the drug constituents [1]. The offending agent interacts directly with bronchial and alveolar cells, which then release T helper-2 (Th2) cytokines, interleukin-5,8,33 (IL-5, IL-8, IL-33), and eosinophils [14-16]. Drugs known to cause eosinophilic pneumonia accumulate in the alveoli leading to epithelial injury and inflammation [2]. Solomon and Schwarz proposed five criteria for the diagnosis of AEP; these include the presence of acute, simple, or chronic eosinophilic pneumonia, the presence of a potential drug, ruling out of other causes, a clinical improvement on the cessation of the drug, recurrence of the disease on the drug being reintroduced [1]. Our patient fulfilled the criteria. The clinical features of AEP include an acute disease characterized by nonproductive cough, dyspnea, fever, malaise, and night sweats, along with peripheral blood eosinophilia, BAL eosinophilia, or eosinophilic infiltration of the lung tissue, as observed on a biopsy [5-8]. Additionally, the patient presented with bilateral patchy areas of ground-glass opacities, interlobular septal thickening, centrilobular nodules [17,18], and bilateral pleural effusion. These infiltrates usually resolve within days of starting corticosteroid treatment [7]. A diagnosis of pneumomediastinum is mainly based on radiology. On a chest radiograph, the presence of subcutaneous emphysema, air around the pulmonary vein, and a continuous diaphragm sign indicated pneumomediastinum [10]. There is not enough literature on any causal association between pneumomediastinum and acute eosinophilic pneumonia. Maybe a drug-induced hypersensitivity reaction is causing bronchial constriction, airway injury, and increased alveolar pressures, ultimately leading to alveolar rupture and pneumomediastinum [9]. Treatment of pneumomediastinum is mostly supportive, focusing on the removal of subcutaneous emphysema with high-flow oxygen delivery and pain management, possibly from the air under the skin [9]. The patient's presentation was suggestive of acute infective exacerbation of bronchial asthma leading to pneumomediastinum. Only a thorough and accurate medical history could exclude asthma and disclose the history of drug consumption. Unfortunately, due to the drug's association with a non-conventional alternative medical regime, it was not possible to definitively identify the causative agent or chemical. The case's rarity lies in its short exposure history, resulting in two separate clinical conditions appearing simultaneously and rapidly resolving with appropriate treatments.

## Conclusions

This case highlights the potential dangers of consuming non-conventional alternative medicines where the chemical contents of the medicine are unknown. The patient received these medicines, from a local practitioner, in a packet with no label mentioning the contents of the medicine. Since non-conventional alternative medicine is a mix of herbal medicines, they rarely mention the chemical components of their drugs, it is thus difficult to ascertain the exact nature of the medicine taken. After ruling out all other possible differential diagnoses and as the patient had not taken any other medicine apart from the non-conventional alternative medicine, it was concluded that the patient experienced acute eosinophilic pneumonia as a result of a hypersensitivity reaction to the non-conventional alternative medicine. Pneumomediastinum represents a very unusual complication of this presentation, and the patient has had no other underlying lung condition, on presentation or in the past, to give rise to the same. To our knowledge, this is the first case report of a spontaneous pneumomediastinum associated with drug-induced acute eosinophilic lung disease. Acute eosinophilic pneumonia could thus be an important differential in patients presenting with pneumomediastinum. Early diagnosis and prompt withdrawal of the offending agent, along with corticosteroid therapy, led to a rapid resolution of symptoms and radiographic abnormalities. This case emphasizes the importance of taking a thorough history, especially when inquiring about drug history. Since most non-conventional alternative treatment regimes rarely disclose the chemicals present in their drugs, the risk of adulteration of such medications increases, which can then lead to serious health risks, as we have seen in our patient.
